# A *Vivens Ex Vivo* Study on the Synergistic Effect of Electrolysis and Freezing on the Cell Nucleus

**DOI:** 10.1371/journal.pone.0145133

**Published:** 2015-12-22

**Authors:** Franco Lugnani, Fabrizio Zanconati, Thomas Marcuzzo, Cristina Bottin, Paul Mikus, Enric Guenther, Nina Klein, Liel Rubinsky, Michael K. Stehling, Boris Rubinsky

**Affiliations:** 1 Franco Lugnani MD, Trieste, Italy; 2 UCO Anatomia ed Istologia Patologica, University of Trieste, Trieste, Italy; 3 Inter Science GmbH, Biophysics, Luzern, Switzerland; 4 Institut fuer Bildgebende Diagnostik, R&D, Offenbach, Hessen, Germany; University of Zurich, SWITZERLAND

## Abstract

Freezing—cryosurgery, and electrolysis—electrochemical therapy (EChT), are two important minimally invasive surgery tissue ablation technologies. Despite major advantages they also have some disadvantages. Cryosurgery cannot induce cell death at high subzero freezing temperatures and requires multiple freeze thaw cycles, while EChT requires high concentrations of electrolytic products—which makes it a lengthy procedure. Based on the observation that freezing increases the concentration of solutes (including products of electrolysis) in the frozen region and permeabilizes the cell membrane to these products, this study examines the hypothesis that there could be a synergistic effect between freezing and electrolysis in their use together for tissue ablation. Using an animal model we refer to as *vivens ex vivo*, which may be of value in reducing the use of animals for experiments, combined with a Hematoxylin stain of the nucleus, we show that there are clinically relevant protocols in which the cell nucleus appears intact when electrolysis and freezing are used separately but is affected by certain combinations of electrolysis and freezing.

## Introduction

Surgery is the field of medicine that employs manual and instrumental techniques to treat medical conditions. Minimally invasive and non-invasive surgery have emerged as an important branch of surgery. They employ a variety of biophysical based techniques, each with their specific advantages, disadvantages and applications. Enlarging the armamentarium of minimally invasive surgery technologies can provide new tools for treating diseases. This study introduces and explores a possible new tissue ablation technique that combines two biophysical processes, freezing and electrolysis. It is interesting to note that both processes originate from the early 19^th^ century work of Faraday on technologies for lowering temperature and on technologies for generating electrochemical reactions, respectively.

Tissue ablation by freezing, currently known as cryosurgery, is used since the middle of the 19^th^ century [1] and employs various physical phenomena and devices to reduce the temperature of the tissue at the points at which a cryosurgery device contacts the tissue. This freezes the tissue in the vicinity of the point of contact. Freezing destroys cells under certain conditions. The group of Gage and Baust has published several comprehensive reviews on all the aspects of the field of cryosurgery including details on the mechanisms of cell death during cryosurgery [2–6]. Freezing changes all the physical properties of tissue. This change in properties facilitates the use of all the known medical imaging technologies, to image the extent of freezing non-invasively and in real time [7,8]. The ability to image the extent of tissue freezing is one of the major advantages of cryosurgery, because it allows real time control over the procedure. However, while medical imaging produces high quality details on the frozen lesion, the extent of cell death does not necessarily coincide with the extent of freezing; in particular on the outer rim of the frozen lesion in the range of higher temperatures and in the vicinity of large blood vessels. Substantial efforts are made to overcome this limitation. These efforts are also extensively reviewed in [2–6].

Electrolysis is also used for tissue ablation since the early 1800’s [9]. The field has experienced a revival in the mid 1970’s with the work of Nordenstrom [10,11]. Electrolytic ablation, also known as Electro-Chemical Therapy (EChT), is a tissue ablation technique that employs products of electrolysis for cell ablation. In EChT electric current is delivered to the treatment field through electrodes that are in contact with the tissue. New chemical species are generated at the interface of the electrodes and tissue as a result of the electric potential driven transfer between electrons and ions or atoms in the tissue. The various chemical species produced near the electrodes diffuse away from the electrodes into tissue, in a process driven by differences in electrochemical potential. Tissue ablation is caused by two factors: the cytotoxic environment developing due to local changes in pH, as well as the presence of some of the new chemical species formed during electrolysis. Electrolytic ablation requires very low direct currents (tens of mA) and very low voltages (single to low tens of Volts) [12]. This is advantageous, because the technology is extremely simple. However, the procedure is long, from tens of minutes to hours. The length is related to the diffusion of electrochemically produced species in tissue and the concentration dependent rate of cell death inducing electro-chemical reactions. A clinical study on tissue ablation with electrolysis states that—“Currently, a limitation of the technique is that it is time consuming” [13,14].

Freezing of tissue increases the concentration of solutes around cells by removing water from solution in form of ice [15]. Freezing also disrupts the cell membrane lipid bilayer and permeabilizes it [16]. We have used these properties to develop a technology for enhancing cell death under freezing conditions in which cells survive cryosurgery. Injecting low concentrations of bleomycin, a cytotoxic compound with limited cell permeability to cells prior to freezing, killed cells at temperatures at which frozen cells normally survive [17]. Control studies have shown that cells survived exposure to these low concentrations of bleomycin in the absence of freezing, and survived freezing without bleomycin. However, they succumbed to the combination [17]. While this combination increased the extent of cell death by cryosurgery, bleomycin is considered a drug, making the procedure complicated due to the constrains on the use of a drug.

As mentioned earlier, one of the limitations of electrolytic ablation is that the procedure requires high concentration of electrolytic products. The need for high concentration of products and the slow diffusion of the products into tissue is what makes the procedure time consuming. It occurred to us that combining electrolysis with freezing will have a similar effect on cell ablation as the combination of freezing with bleomycin, without the need to inject a drug. The idea is based on the two properties of freezing discussed in the previous paragraph, concentration of solutes and permeabilization of the cell membrane. In this study we examine the hypothesis that a combination of electrolysis and cryosurgery can produce more extensive cell death than the same electrolytic ablation or cryosurgery ablation procedure alone. The rationale for the hypothesis is that when electrolysis is performed prior to freezing, freezing would concentrate the products of electrolysis around a cell and permeabilize the cell membrane to expose the intracellular space to these products. This combination would be particularly valuable in freezing regimes in which cells survive freezing and amounts of electrolytic products in which cells survive electrolysis.

This study also has a secondary goal, which we, nevertheless, consider as important. It illustrates the value of a research model named “*vivens ex vivo”*. The inspiration for this model is the 3R principles for animal care (Replacement, Reduction, Refinement) of Russell and Burch [18]. As mentioned earlier, this study is a first order examination of the hypothesis that the combination of electrolysis and cryosurgery can produce more extensive cell death than electrolysis or cryosurgery separately. Animal research is needed, because currently available methods for cell work and mathematical modeling cannot capture all the complexity of organized tissue. However, we thought that at an early stage of examining a hypothesis, it should be possible to gain insight into the value of the hypothesis without using a live animal for the experiment. Therefore, in this study we found a way to use fresh animal tissue whose provenance is animals which died from reasons not related to the study. We generated an experimental set-up that allowed us to examine pig liver tissue, from a commercial abattoir for food (Sloughtershouse EE Aurisina Trieste Italy), within 10 minutes from the animal death. Because, within this period of time, liver tissue exposed to ischemic conditions does not begin yet to deteriorate, we call the model *vivens ex vivo*. In this study, the *vivens ex vivo* livers were treated with cryosurgery, electrolysis and electrolysis with cryosurgery and the extent of tissue ablation at the cathode and anode was compared.

## Materials and Methods

Liver tissue from 200 kg pigs were treated within 10 minutes from animal death. The livers were obtained from a certified and regulated commercial abattoir and the experiments were carried out at an adjacent location outside the abattoir. Cryosurgery was delivered with a cryosurgery system, Cryo Electric S model CO_2_ driven Metrum Cryoflex Warsaw, Poland using commercial 2 mm, G20 type reusable cryoprobes, Trocar type, Cryoflex Warsaw, Poland. Electrolysis was delivered using a laboratory power supply (HCS 3304 USB, Manson, Hong Kong), whose electric output was attached to steel rods, with the same diameter as the cryosurgery probes, namely 2 mm.

**Fig 1:**
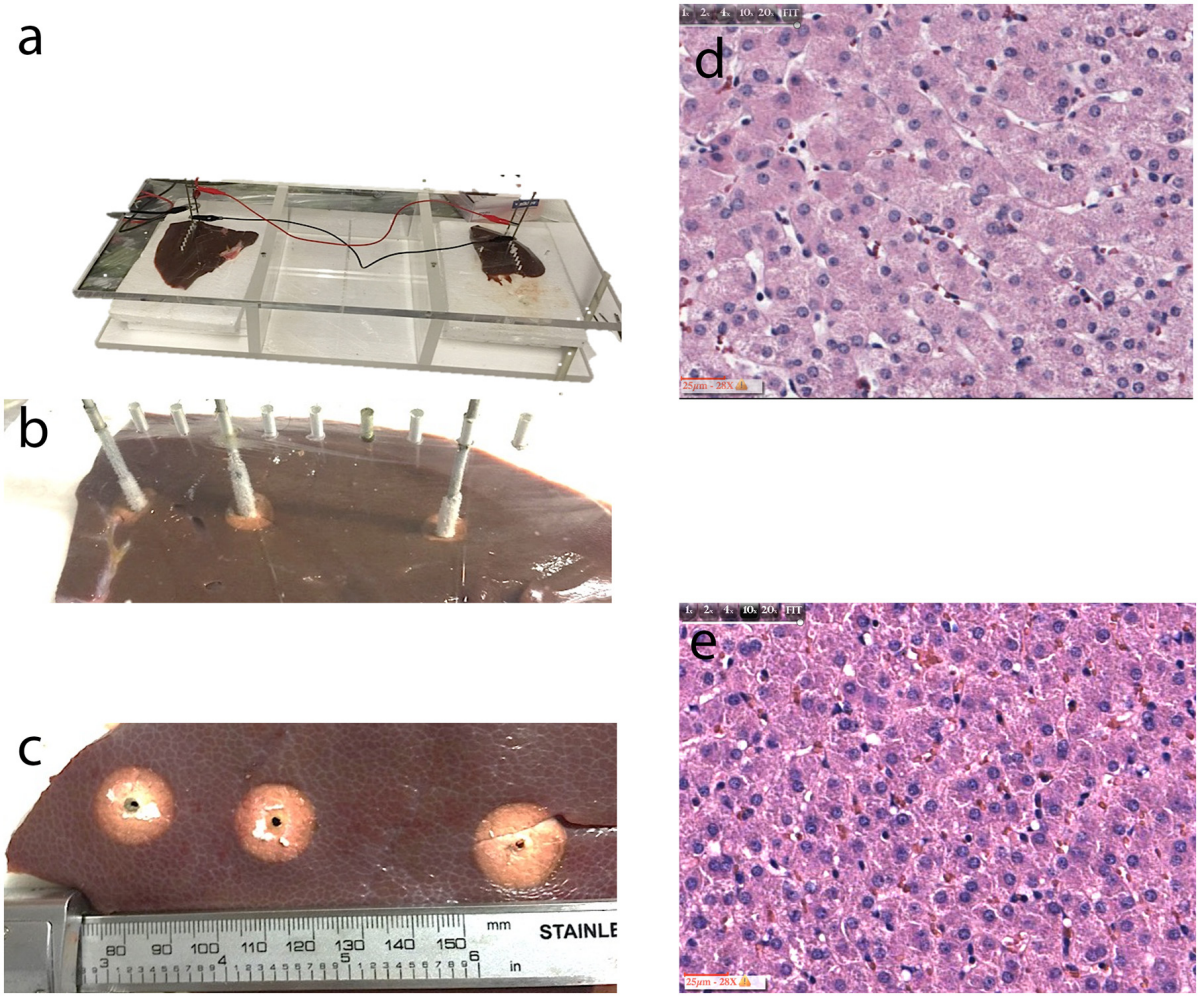
Experimental setup. **a:** a stand for multiple parallel cryosurgery and electrolysis experiments. **b:** simultaneous freezing of three sites in the same liver. The two lesions on the left have experienced electrolysis and now cryosurgery and the left is experiencing only cryosurgery. **c:** appearance of the frozen lesion immediately after freezing. The circular light area is the frozen zone. **d:** untreated *in vivo* pig liver **e:** untreated liver from this *vivens ex vivo* pig liver study.

The experimental setup is shown in Fig 1. The experiment information is given in the left column of panels. The top panel shows the transparent, Plexiglas made setup, which allows for three liver samples to be treated simultaneously. Fig 1 shows two liver samples in their particular setting. An arrangement was made for the electrodes or cryosurgical probes to be inserted vertical into the liver through multiple parallel drilled holes, at distances of 1 cm between each. The arrangement allows for the delivery of equal doses of electricity and cooling, to different samples from the same animal. This particular figure shows the electrolysis electrodes connected in parallel to the power supply. On each liver sample, the cathode and anode were separated by 3 cm. Shown in the middle left side panel is the appearance of an experiment in which, following the delivery of the electrolysis, we replaced on one sample the electrolysis electrodes with two cryosurgery probes. In addition we inserted a third cryosurgery probe at a distance from the sites treated by electrolysis. The three cryosurgery probes froze the tissue until an ice lesion of about 20 mm formed around the probes (Fig 1, panel c). We found that 10 minutes freezing with our cryosurgery system generates a 20 +/-1 mm diameter lesion. An experiment of the kind illustrated in these panels generates the following samples: a) electrolysis only at the anode, b) electrolysis only at the cathode, c) electrolysis at the anode followed by 10 minutes freezing, d) electrolysis at the cathode followed by 10 minutes freezing, e) 10 minutes freezing only. This allowed us to compare, in the same liver and with identical electrical and thermal parameters, the outcome: of electrolysis only, of freezing only and of the combination. For electrolysis we applied DC current set to 100 mA for 5 minutes and for 10 minutes. Three repeats were done for each condition. The samples were fixed in formalin embedded in paraffin and the slides stained with Hematoxylin and Eosin (H&E). The stained samples were scanned with a digital microscope D-Sight Fluo 2.0 (A. Menarini, Diagnostics, S.r.l, Firenze, Italy) in preparation for histological examination.

The histological examination compared the morphology of cells in the treated areas with that of cells in untreated hepatic tissue. The examination has focused primarily on changes in the nucleus, which is a more stringent criteria of cell damage than the cytoplasm and cell membrane. The nucleus is particularly appropriate for the *vivens ex vivo* tissue model. The cell membrane could begin to experience some morphological changes due to ischemia after animal death and prior to the ablation treatment. Morphological changes to the nucleus occur much later after animal death. Therefore we will focus primarily on the Hematoxylin stain of the nucleus. Samples from the same slide and at a site remote from the treated area were taken to represent untreated tissue and used for comparison.

In addition to the qualitative analysis of the histological samples, we performed a quantitative analysis of the structural changes of the nuclei by evaluating the nuclei surface area. We scanned the Hematoxylin-Eosin stained slides with the D-Sight Fluo 2.0 scanner and its attendant image measurement software (VISIA Imaging S.r.l., Valdamo, Italy). Of every slide, we scanned the area near the probe insertion with a 60x magnification on bright field and oil submersion. Inside the acquired image we selected a single area of 0.11 mm2 (equivalent to a 60x magnification) where the tissue damage was evident. The selected area was chosen to contain only hepatocytes, avoiding stromal or inflammation zones. Inside this analysis area the surface area of every hepatic cell nucleus was measured with the dedicated software function. Only nuclei with clearly detectable nuclear membrane were counted. Nuclei that were cut by the edge of the selected area were not taken into consideration. A normal area, selected far away from the area of the probe insertion and assessed by an expert pathologist as normal, was used as the control. Results were recorded in an Excel document and analyzed calculating the mean of the data of every slide and the standard deviation and comparing it with the control.

## Results

The two panels on the right hand side on Fig 1 were used to establish the base line for the histological evaluation. The top panel shows the cellular morphology of pig liver tissue from an *in vivo* study. The bottom panel shows the cellular morphology of a *vivens ex vivo* pig liver from an area which we used as control. With regards to the nucleus, it has a similar morphology in both panels. The nuclei are transparent and round, with normal chromatin distribution, and the nucleolus remain clearly visible.

**Fig 2:**
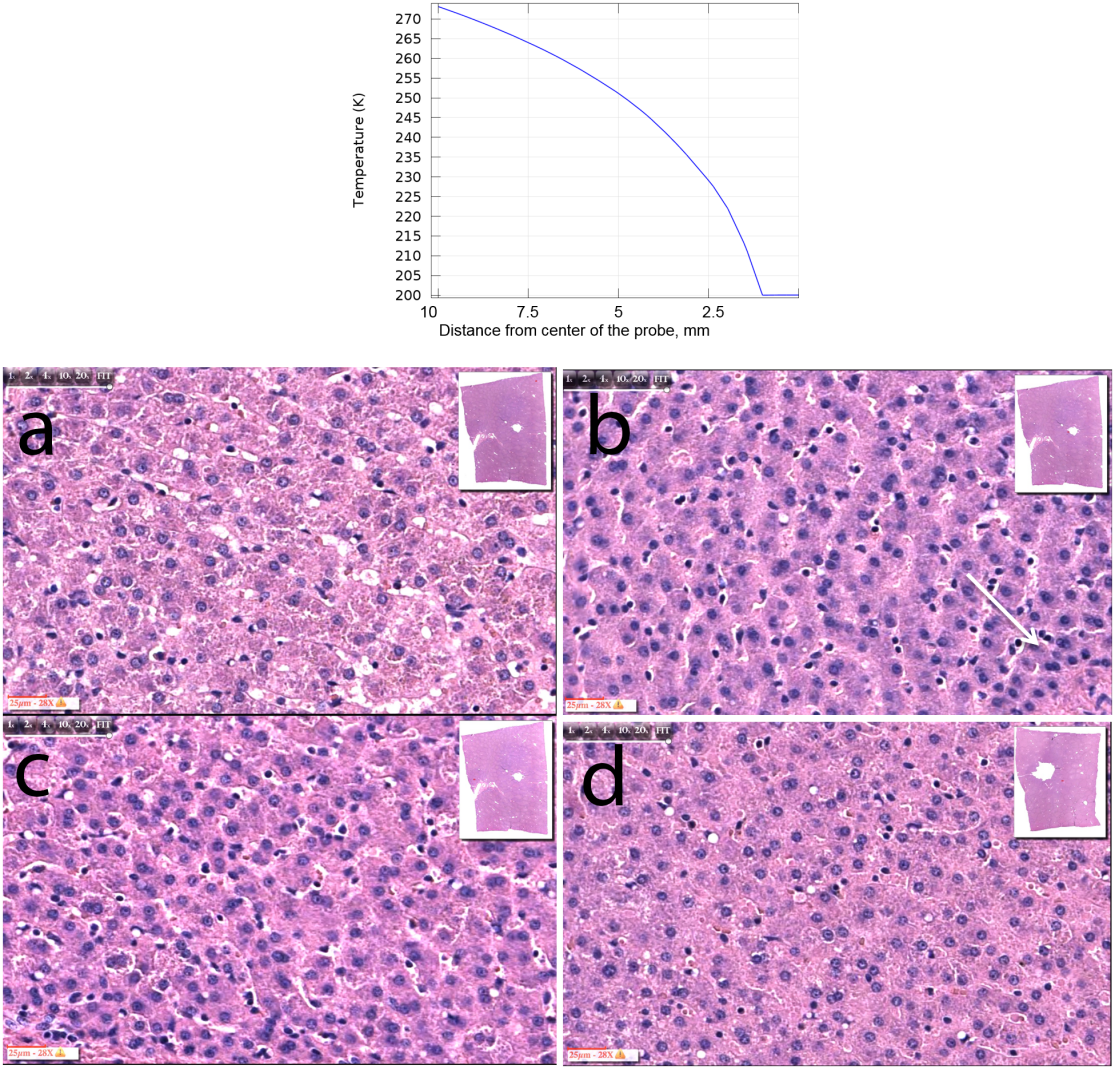
Freezing only at different distances from probe. **Top schematic:** Temperature distribution from center of the probe; calculated lower limit. Micrograph panels show images from liver tissue treated by freezing only. **a:** control, **b:** near probe (probe edge on right), **c:** 10 mm from probe center, **d:** 5 mm from probe center (taken from another experiment to illustrate consistency among repeats). Insert shows site from which the panel was taken.

Fig 2 illustrates the effect of 10 minutes freezing on the liver. The top panel shows the calculated temperature distribution the from center of the probe. The temperature distribution was obtained from the solution of the steady state Laplace equation for a boundary condition of -79°C on the cryosurgery probe and 0°C on the outer edge of the ice lesion. This represents the so-called quasi steady state solution of the temperature distribution in frozen tissue [19]. The analysis was done with Comsol Multiphysics 4.2 (Comsol, Burlington, MA). The temperature profile is the lower limit of the possible temperature distribution. In our microscopic analysis we have taken treated samples from three sites: near the probe +/- 1 mm; at a diameters of 10 +/- 1mm, and at a diameter of 20 +/- 1mm (within the frozen region). The approximate temperatures, that the liver tissue at those sites experienced are, respectively: -70°C; -15°C and -2°C. This estimate is very rough and should be viewed as an order of magnitude estimate. Micrograph panels show clockwise from top left: control (panel a), near probe (panel b) (probe edge on right), roughly 5 mm from probe center (panel d) (taken from another experiment to illustrate consistency among repeats), roughly 10 mm from probe center (panel c). Insert shows the entire tissue sample and the site from which the magnified image was taken. Focusing on the nucleus, it is evident that in all the panels the nuclei are fully developed, round and transparent, with normal chromatin distribution, and a clearly visible nucleolus. There are very few cells, just adjacent to the probe, in which some of the nuclei have become pyknotic. They are marked with an arrow. The damage is limited to only very few cells, while the great majority of the hepatocytes are morphologically healthy.

**Fig 3:**
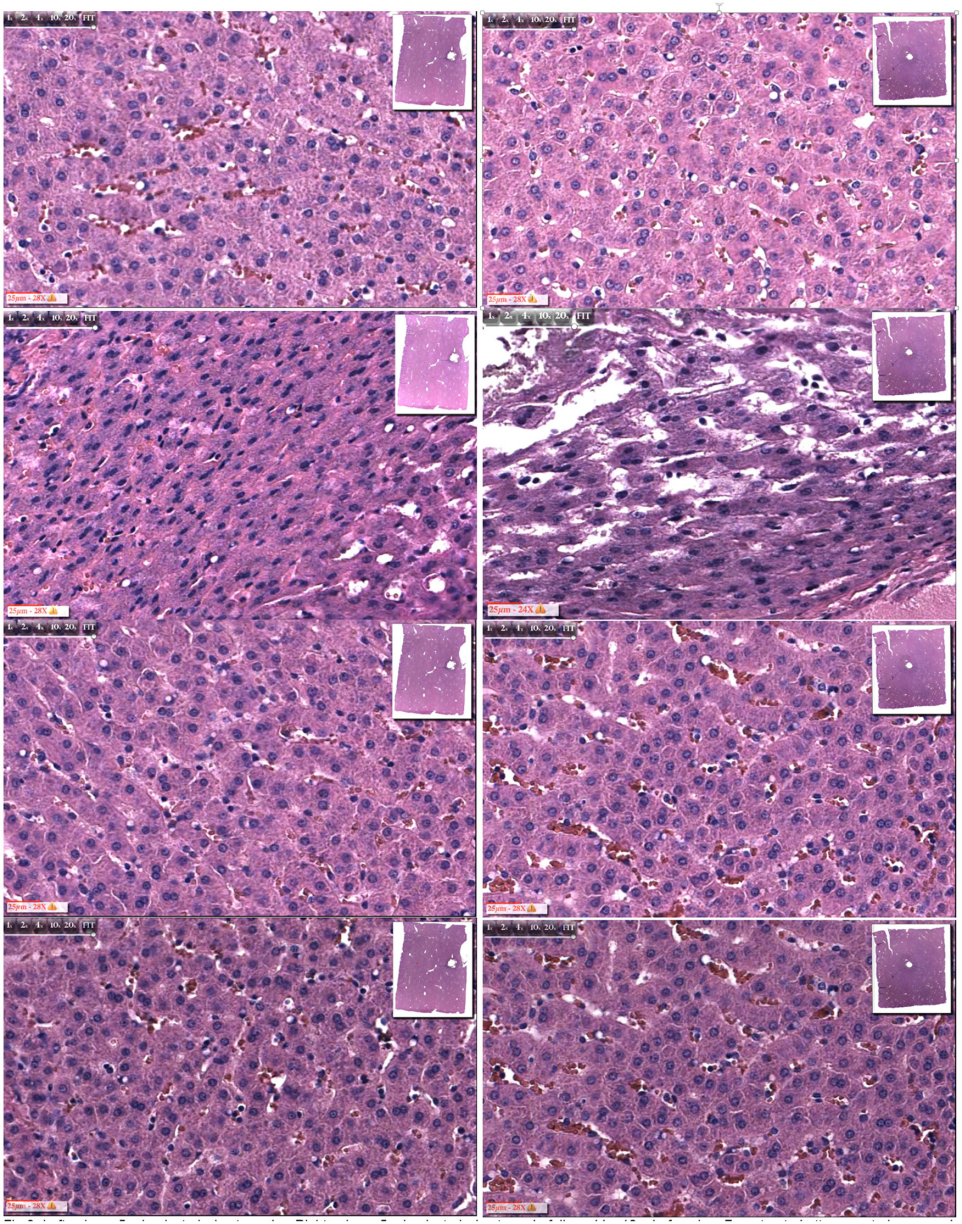
5 minutes only electrolysis at the anode vs. 5 minutes electrolysis plus freezing. Left column: 5 minutes electrolysis at anode; right column: 5 minutes electrolysis at anode followed by 10 minutes freezing. From top to bottom: control; near probe; 5 mm from center of probe; 10 mm from center of probe.

**Fig 4:**
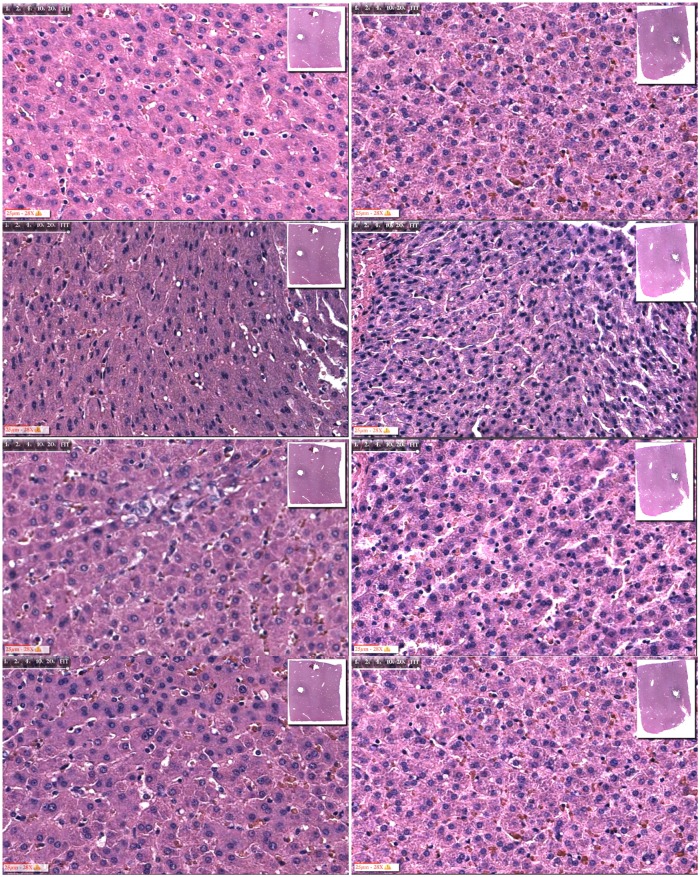
10 minutes only electrolysis at the cathode vs. 10 minutes electrolysis plus freezing. Left column: 10 minutes electrolysis at anode; right column: 10 minutes electrolysis at anode followed by 10 minutes freezing. From top to bottom: control; near probe; 5 mm from center of probe; 10 mm from center of probe.

**Fig 5:**
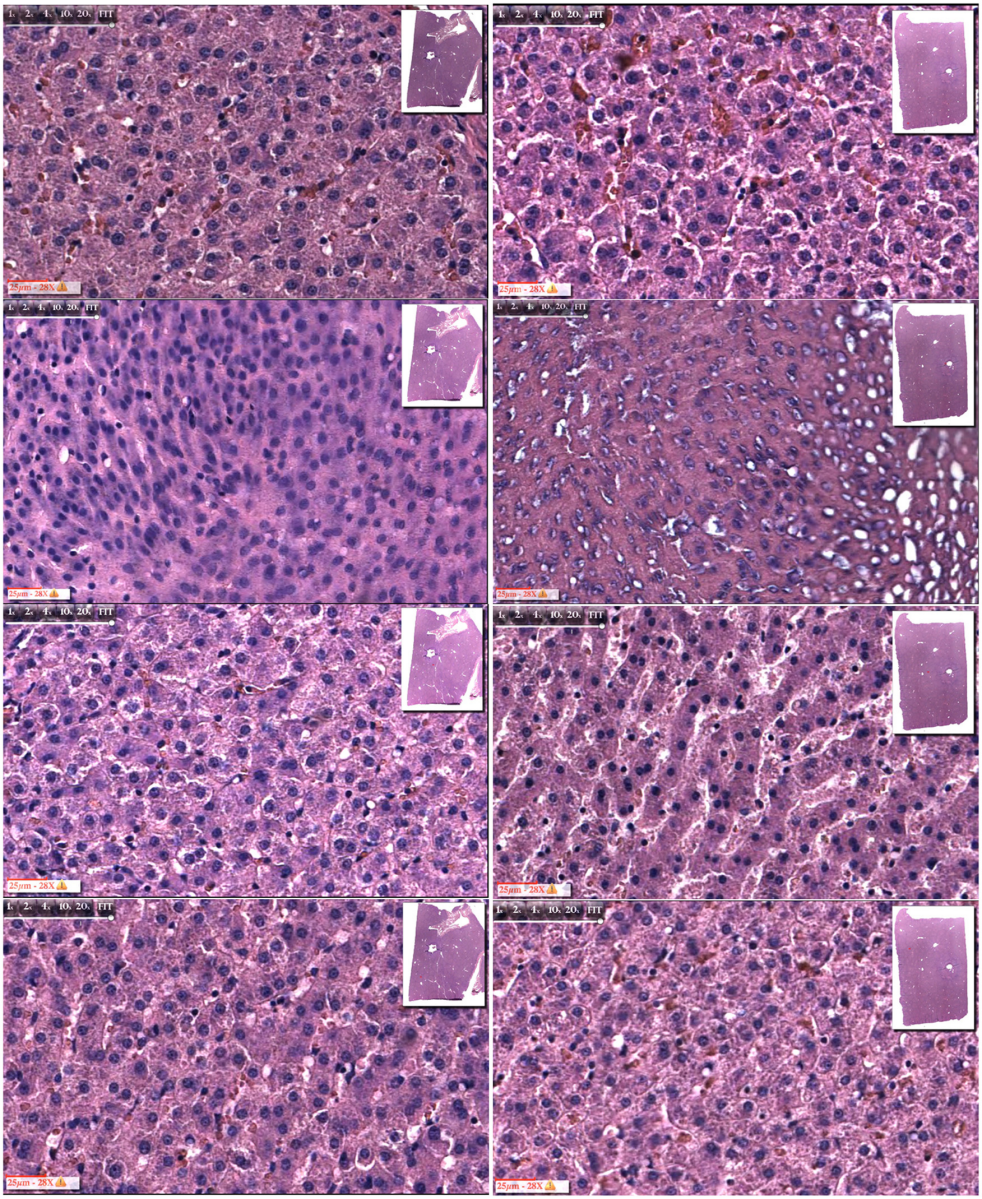
5 minutes only electrolysis at the anode vs. 5 minutes electrolysis plus freezing. Left column: 5 minutes electrolysis at cathode; right column: 5 minutes electrolysis at cathode followed by 10 minutes freezing. From top to bottom: control; near probe; 5 mm from center of probe; 10 mm from center of probe.

**Fig 6:**
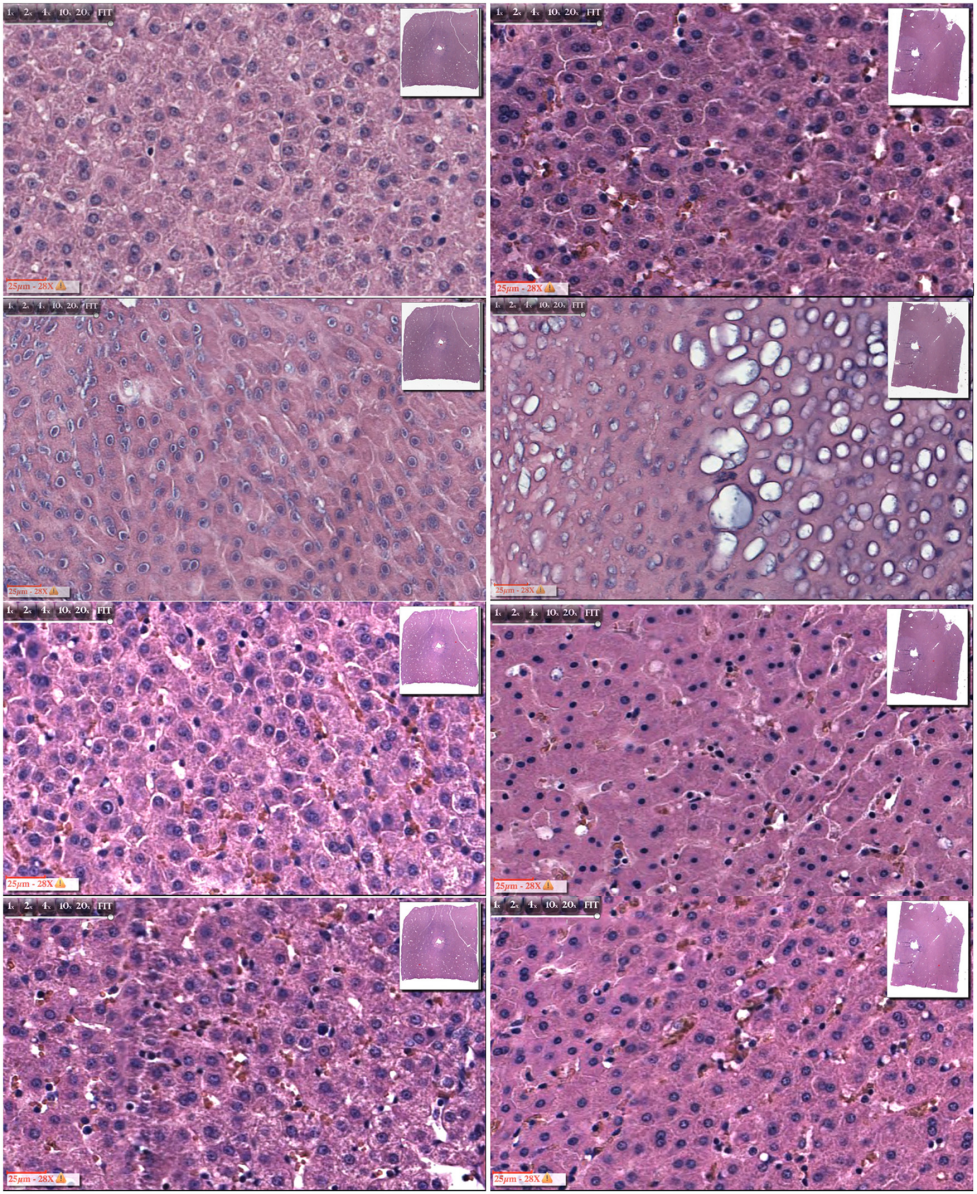
10 minutes only electrolysis at the cathode vs. 10 minutes electrolysis plus freezing. Left column: 10 minutes electrolysis at cathode; Right column: 10 minutes electrolysis at cathode followed by 10 minutes freezing. From top to bottom: control; near probe; 5 mm from center of probe; 10 mm from center of probe.

The format of Figs 3 to 6 is similar. The left column of panels is for electrolysis alone and the right column is for electrolysis followed by 10 minutes of freezing to a diameter of about 20 mm. The rows of panels are for magnified microscopic images. They are, from top to bottom: control; near the probe +/- 1 mm; at a diameters of 10 +/- 1 mm, and at a diameter of 20+/- 1 mm (within the frozen region). The possible temperatures that the frozen liver tissues at those sites experienced are, respectively: -70°C; -15°C and -2°C. This estimate is very rough and should be viewed as an order of magnitude estimate.

The effect of 5 minutes electrolysis at the anode is examined in Fig 3. The morphology of the cells at all the treatment sites is similar to the controls, except adjacent to the probe. Near the anode, electrolysis produces substantial damage. The second left side panel from top illustrates the damage. The process of electrolysis causes a distortion and shrinkage of the nucleus, which becomes pyknotic. The nucleolus has disappeared and there is an increase in nuclei polymorphism. The chromatin has lost its homogeneous distribution. It is interesting to notice that the distortion of the nucleus has a directionality. We found this in all the three repeats. The additional effect of freezing, shown in the second right panel from top, produces a similar effect on the nuclei. In addition there seems to be damage to the macroscopic architecture of the tissue, with tears between cells.

The effect of 10 minutes electrolysis at the anode is examined in Fig 4. The morphology of the cells at all the treatment sites is similar to the controls, except adjacent to the probe. Near the anode, electrolysis produces substantial damage. The second left side panel from top illustrates the damage. The process of electrolysis causes a distortion and shrinkage of the nucleus, which becomes pyknotic. The nucleolus has disappeared and there is an increase in nuclei polymorphism. The chromatin has lost its homogeneous distribution. Again, it is interesting to notice that the distortion of the nucleus has a directionality. The results shown here are similar to those in Fig 3. We found this in all the three repeats. The additional effect of freezing is shown in the second right panel from top. In addition to an effect on the nucleus similar to that in the left hand side panel, there seems to be damage to the macroscopic architecture of the tissue, with tears between cells. The third row from the top shows microscopic images taken from sites at about 10 mm diameter around the center of the probe. The left hand panel shows that in the absence of freezing the cell morphology remains intact and similar to the controls. The right hand side panels shows, inconclusively, that when the tissue is frozen after electrolysis that some nuclei appear to have shrunk relative to the controls. However, these images are inconclusive and cannot be used as evidence that the hypothesis of this study is correct.

The effect of 5 minutes electrolysis at the cathode is examined in Fig 5. Electrolysis alone produces substantial damage near the cathode, as seen in the second panel from top, left column. However, the effects are different from those near the anode at the same relative location. The nuclei are swollen, with thickened chromatic in the center and with a perinuclear ring. They are larger than in normal tissue and distorted. Nuclear polymorphism is present. Swelling of the cell cytoplasm was also observed. The left column shows that all the other examined sites were not affected by electrolysis alone. The right column shows that near the cathode, the combination electrolysis and freezing, substantially increases the damage relative to electrolysis or freezing alone. Near the electrode there is evidence of much more severe damage than from electrolysis alone. There is severe damage to the cytoplasm and nuclei, which present macro-vacuoles. The nuclei are pyknotic and polymorphic. The third row from the top compares tissue samples from a diameter of about 10 mm around the cathode in which the tissue was treated with electrolysis alone, left panel, and in which the tissue was treated by electrolysis followed by freezing, right panel. The left panel shows intact cells and nuclei. In contrast, the nuclei in the right panel are pyknotic and polymorphic. Obviously, here, tissue treated by the combination of electrolysis and freezing has a different morphology from tissue treated by electrolysis alone (left panel) or tissue treated by freezing alone (Fig 2).

The effect of 10 minutes electrolysis at the cathode is examined in Fig 6. Electrolysis alone produces substantial damage near the cathode, as seen in the second panel from top, left column. The nuclei are swollen, with thickened chromatic in the center and with a perinuclear ring. There is severe damage to the cytoplasm and nuclei, which present vacuoles. They are larger than in normal tissue and distorted. Nuclear polymorphism is present. Swelling of the cell cytoplasm was also observed. The left column shows that all the other examined sites were not affected by electrolysis alone. The right column shows that near the cathode, the combination electrolysis and freezing, substantially increases the damage relative to electrolysis or freezing alone. Near the electrode there is evidence of much more severe damage than from electrolysis alone. There is severe damage to the cytoplasm and nuclei, which present macro-vacuoles. The nuclei are pyknotic and polymorphic. There are actual sites, where the cells were completely obliterated. The third row from the top compares tissue samples from a diameter of about 10 mm around the cathode in which the tissue was treated with electrolysis alone, left panel, and in which the tissue was treated by electrolysis followed by freezing, right panel. The left panel shows intact cells and nuclei. In contrast, the nuclei in the right panel are pyknotic and polymorphic. They substantially shrunk and there is no evidence of cell membrane or any intact morphology. Obviously, here, tissue treated by the combination of electrolysis and freezing (Fig 2, right panel) has a different morphology from tissue treated by electrolysis alone (Fig 2, left panel) or tissue treated by freezing alone.

**Fig 7:**
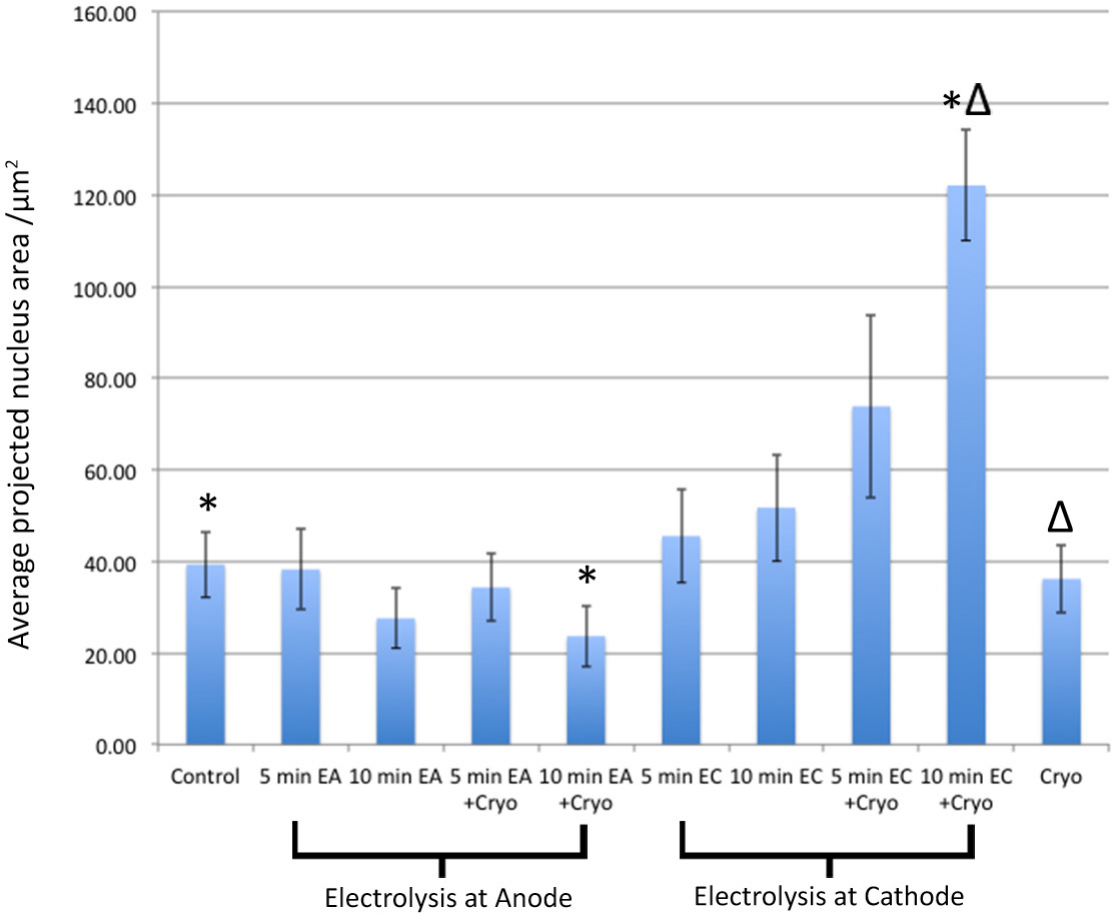
Average surface area of nuclei ±*σ*, near the probe, for various treatment protocols. Time listed indicates the time of electrolysis. EA—electrolysis near anode; EC—electrolysis near cathode; cryo—indicates freezing; *- significantly different compared to control with p<0.05; Δ- significantly different compared to cryo only with p<0.05.

The projected nucleus area of the hepatocytes nuclei was evaluated using a measurement software attached to the scanning microscope (VISIA Imaging S.r.l., Valdamo, Italy). The average projected nucleus area and the standard deviation near the probe is illustrated in Fig 7 for each of the nine experimental conditions in comparison with controls. The histological observations in Figs 2 to 6 are reflected by the quantifiable data in Fig 7. Several conclusions can be made with a statistical significance level of < 0.05. Near the anode, ten minutes of electrolysis followed by cryosurgery causes a shrinkage of the nucleus in relation to both a normal nucleus and to the nucleus in tissue treated by cryosurgery alone. Near the cathode, ten minutes of electrolysis followed by cryosurgery causes an expansion of the nucleus relative to a normal nucleus and relative to a nucleus in tissue treated by electrolysis alone or by cryosurgery alone. It is also interesting to notice that when 10 minutes of electrolysis are delivered alone, the nucleus near the cathode is expanded relative to the nucleus near the anode with a statistically significant p value of < 0.05.

## Discussion

Obviously, our analysis is limited by the nature of the model we have used. Nevertheless, we believe that there is value in using the *vivens ex vivo* model in a first feasibility study. In fact it is a requirement of the 3R principle. We believe that this study shows that despite its limitations, the model can generate important information.

Fig 2 shows that the freezing protocol we employ produces limited evidence of cell death. It should be emphasized that we have chosen the freezing protocol for this outcome. Our goal was to examine the hypothesis that the combination electrolysis and freezing produces tissue ablation under conditions in which cell death is not induced by either freezing or electrolysis alone. Many times, cells treated by cryosurgery survive brief, one cycle freezing to -50°C [16]. The calculated temperature distribution shows that the frozen tissue experiences temperatures higher than -50°C already 1 mm near the probe. Furthermore, the temperature profile is a lower limit estimate. It is likely that, because of the heat transfer coefficient in the probe, the temperature distribution in the frozen lesion is substantially higher than the calculated temperature. Therefore, it is not surprising that the cells in frozen lesions appear intact. It should be added that apoptosis is an important mechanism of cell death during cryosurgery [2–6, 20–22]. The mechanism of apoptosis is particularly dominant at the outer edge of the frozen lesion, which this study examines [2–6, 20–22]. However, apoptosis requires an intact metabolic system, which is missing in this model and is one of the major limitations of the model. It is important to note, for the examination of the hypothesis, that at a distance of 5 mm from the center of the probe, freezing has no effect on the nucleus.

Tissue ablation by electrolysis has been studied extensively, and some of the key references can be found in the introduction. It is generally agreed that the primary mechanisms of damage during electrolysis are the toxic species produced by the electrolytic electrochemical reactions. The main electrochemical reactions in a saline solution, are the decomposition of water and the oxidation of chloride. The chemical reaction that takes place is:

$$2\text{NaCl }+{\text{ 2H}}_{\text{2}}\text{O }\to \;2\text{ NaOH }+{\text{ H}}_{\text{2}}\;+{\text{Cl}}_{\text{2}}$$

Chlorine is produced at the anode and hydrogen at the cathode. In the presence of active electrodes such as iron, there are other reactions at the anode, driven by the anode current, such as:

$$\text{F}e_{2}+\text{ aq }\to {\text{ Fe}}_{\text{3}}\;+\text{ aq }+\text{ e}$$

The cathode material is protected against dissolution by the cathodic current and the majority of the products are molecular hydrogen and hydroxyl ions. These are the ablative chemical species at the cathode. At the anode, the chloride reacts with water to form hypochlorous acid and other chlorine based species that are toxic to organic molecules. The chemical species produced at the anode and cathode are transported by diffusion due to concentration gradients and electro migration due to the electric potential gradient. In addition, the electric field causes a flux of water (electro-osmosis) from the anode to the cathode, causing edema near the cathode and the tissue around the anode to dehydrate. It was shown that the pH changes due to electrolysis are also a major cause of cell death.

Figs 3 to 6 show that following electrolysis, there is cell death near the anode and cathode. It is interesting to notice that the appearance of the nucleus is different at the anode from the cathode. There is shrinkage of the nucleus near the anode and swelling near the cathode. This can be explained by the different mechanisms of cell death at the anode and cathode, caused by the different products of electrolysis. Another possible explanation is the electro diffusion of water from the anode to the cathode, with the tissue dehydrations near the anode and swelling near the cathode. Increasing the time of exposure to electrolytic products seems to increase the distortion of the nucleus near the electrodes. However, in all the studies, the damage is localized just adjacent to the probe. For the examination of the hypothesis, it is important to notice that at a distance of about 5 mm from the center of the probe, the nucleus is not affected, even after 10 minutes of electrolysis.

Figs 3 to 6 show that near the electrodes, a combination of freezing and electrolysis produces a different tissue morphology compared to electrolysis or freezing alone. While, near the electrodes, freezing alone produces limited damage and electrolysis produces damage primarily to the nucleus, the addition of freezing to electrolysis enhances the extent of tissue damage. The enhancement relates primarily to the formation of voids in the tissue. We believe that they are caused by the formation of large ice crystals that disrupt the morphology of the tissue which was weakened by electrolysis.

Perhaps the most important results from this study are in Figs 5 and 6, the third panels from the top. It is evident that near the cathode, at a distance of about 5 mm around the cathode, the nucleus appears intact when only freezing or electrolysis are applied separately. In contrast, there is a substantial effect on the nucleus when the combination of freezing and electrolysis is applied. The distortion of the nucleus is larger when the electrolysis was delivered for 10 minutes, than for 5 minutes. In Fig 6, third panel from the top, right hand side, the morphology of the cells has completely disappeared. The synergistic effects of the combination electrolysis and cryosurgery is also observed near the anode. However, the effect is weaker compared to that near the anode. Fig 3, third row from the top, shows that the tissue morphology, at a location 5 mm from the center of the probe, is the same for the controls, the combination of 5 minutes electrolysis and 10 minutes freezing, 5 minute electrolysis alone and 10 minutes freezing alone (Fig 2). In contrast, Fig 4, third row from the top shows that, at a location 5 mm from the center of the probe, the nucleus is affected by the combination of 5 minutes electrolysis and 10 minutes freezing, while it remains similar to the controls for 5 minute electrolysis alone or 10 minutes freezing alone (Fig 2).

The results in Fig 7 provide some quantification to the histological observations. We have made several additional measurements, such as the perimeter of the nucleus. However, the surface area provides more compelling values. With respect to the surface area of the nucleus, there is no statistically significant difference between the controls and tissues treated by cryosurgery alone or by 5 minutes or 10 minutes electrolysis alone. It is interesting to notice that near the anode, 10 minutes of electrolysis causes the nucleus to shrink. While the shrinkage is not yet statistically significant, the shrinkage seems to occur. Qualitative morphological observations show that for 10 minutes of electrolysis near the anode, the whole tissue seems to be wrinkled and the nuclei take elliptical elongated shape. However, adding cryosurgery after electrolysis causes an additional shrinkage of the nucleus near the anode, to a level that becomes statistically relevant. The mechanism is probably the migration of water from the anode to the cathode, which is a well established phenomenon in electrolysis.

The changes in the nucleus near the cathode are different from those near the anode. Both 5 minutes of electrolysis and 10 minutes of electrolysis followed by cryosurgery cause a substantial swelling of the nucleus near the cathode, relative to the controls and cryosurgery or electrolysis alone. Indeed, near the cathode the nuclei are very large with distinct nuclear vacuolization. While the expansion near the cathode is much larger than the shrinkage near the anode, the difference is probably caused by the inherent properties of a cell with respect to it’s ability to shrink or expand and not because cell damage is more extensive at the cathode than the anode. In summary, the quantitative analysis shows that the combination of cryosurgery and electroporation produce a statistically significant change in the nucleus, while cryosurgery and electroporation separately do not produce such a change

The results of this study could be considered a first order proof of our hypothesis. Using a *vivens ex vivo* model and Hematoxylin stained nucleus as a morphological evaluation criteria, we found that there are circumstances in which cells (the nucleus) are affected by a combination of electrolysis and freezing while electrolysis and freezing alone, with the same parameters, do not affect the nucleus. The mechanism may relate to the effect of freezing on concentrating the solutes around cells and on opening access to the interior of the cells for the toxic products of electrolysis. These toxic compounds are more effective at inducing cell death, when the cell membrane is open and they can access the nucleus. Obviously the toxic effect is a function of concentration, which is higher and diffuses farther away the longer the application of the electrolytic current.

The parameters we have examined in this study are relevant to clinical applications. Freezing was done at temperatures in which cells normally survive clinical procedures and electrolysis was done for a dose in which cells also normally survive clinical procedures. The combination causes cell death, which suggests that it may have clinical value to enhance cell ablation.

Obviously substantially more research needs to be done following this preliminary first order study. However, the value of this study is that it shows in a *vivens ex vivo* tissue model that the combination of electrolysis and cryosurgery is more effective at tissue ablation than either electrolysis or cryosurgery alone and it suggests a simple way to make these tissue ablation technologies more effective.
